# Rhodamine-immobilized optical hydrogels with shape deformation and Hg^2+^-sensitive fluorescence behaviors

**DOI:** 10.1038/s41598-020-64549-5

**Published:** 2020-05-07

**Authors:** Zixiang Qu, Xia Meng, Hongdong Duan, Dawei Qin, Lizhen Wang

**Affiliations:** 1 School of Chemistry and Pharmaceutical Engineering, Qilu University of Technology (Shandong Academy of Sciences), Jinan, Shandong Province 250353 China; 2 Biology Institute, Qilu University of Technology (Shandong Academy of Sciences), Jinan, Shandong Province 250014 China

**Keywords:** Environmental sciences, Chemistry, Materials science

## Abstract

A highly effective method for the research and development of a novel macroscopic hydrogel sensor and bilayer hydrogel is reported. Based on Rhodamine 6G, an Hg^2+^ sensitive fluorescent functional monomer was synthesized, then the monomer was utilized to synthesize hydrogel sensors and bilayer hydrogels. Hydrogel sensor has prominent selectivity to Hg^2+^, the bilayer hydrogel has shape changing function additionally. By combining a thermoresponsive hydrogel layer, poly N-isopropylacrylamide (PNIPAM), with an Hg^2+^ selective hydrogel layer via macroscopic supramolecular assembly, a bilayer hydrogel is obtained that can be tailored and reswells. The bilayer hydrogel sensor can show complex shape deformation caused by the PNIPAM layer and the Hg^2+^-responsive characteristic of hydrogel sensor layer can be observed under visible light or UV light. This work will provide novel insights for the design and synthesis of novel smart materials with synergistic functions.

## Introduction

Macroscopic supramolecular polymer hydrogel networks are excellent candidates for smart sensing materials. Many kinds of synthetic hydrogels have been used for smart material applications because of their functionalization during synthesis in comparison with their natural hydrogel counterparts of polysaccharides and proteins^1^. Furthermore, most hydrogels are transparent and stretchable, with low cost and free environmental pollution. Especially, hydrogels that are characterized by recognizing and responding specific heavy metal ions are promising in the development of sensors for manipulation in heavy-metal-polluted environment^2^.

It has been proved that Mercury (II) is one of the most widely existing toxic heavy metal ions and plays critical physiological roles in organisms due to the damage of biological systems by Hg^2+^ ion inside in the way of disrupting biological processes, which results in serious diseases, including brain damage, kidney failure, all kinds of motion and cognitive disorders. Hydrogel systems, with Hg^2+^ responsive characteristic, can be functioned as the microactuator that is capable to be manipulated in Hg^2+^ polluted microenvironment^3,4^. Therefore, the development of hydrogel systems is not only a rapid, convenient and efficient method that can detect Hg^2+^ ion but also is of both scientific and technological importance^5^.

Recently, composite materials that based on macroscopic supramolecular hydrogels were investigated for optical detection as well as the removal of diverse metal ions^6^. Up to now, a vast variety of synthetic strategies of hydrogel sensors have been developed and studied, accompanying by their functionalization procedures, performance control and potential applications^7^. In particular, the synthesis of equipment-free hydrogel sensor systems merely through colorimetric and fluorescent detection by the naked eye is not only feasible, but will also be of the most profound significance and widest practicability on account of simple manipulation, excellent sensitivity and high signal-to-noise ratio^8,9^. Meanwhile, these materials are easily prepared with low-cost^10^, which are convenient to utilize in various conditions^11^.

However, compared with living organisms in nature, the currently investigated sensing hydrogels can simply deform in manners of swelling/shrinking, as integrating color-changing and complex shape deformation abilities in one system is challengeable^12^. Aiming at expanding the utility and functionality of polymeric hydrogel sensors, polymeric hydrogel sensors, which can respond and transfer various multiple external stimuli to controllable mechanical deformations such as swelling, shrinking, bending and buckling^13,14^, shall be hereby broadly applied in switches, microrobots, artificial muscles, motors and other new applications^3,15,16^.

In this study, a transparent hydrogel sensor that shows fluorescence in response to sensing Hg^2+^ was reported, and based on this hydrogel sensor, a bilayer hydrogel was synthesized that characterized by Hg^2+^-response and shape-changing. When exposed to Hg^2+^, the color of the hydrogels turned to intensive red and the color intensity was associated with the Hg^2+^ ions concentration. Using the ethylenediamine (EDA) solution, we investigated the reusability of hydrogel sensor. Aiming at integration of properties of fluorescence color-changing and shape deformation into one hydrogel system, we synthesized a bilayer hydrogel by the hydrogel sensor and PNIPAM. In generally, the hydrogel sensor features selective, reversible sensing of Hg^2+^ and the bilayer hydrogel integrates Hg^2+^-sensing and shape-changing into one system. The work provides an easy-using and intelligent sensing system, which can be reused for many times with multiple synergistic functions in aqueous environments.

The synthesis of hydrogel sensors and bilayer hydrogels will bring numerous opportunities in a wide range of applications in luminescent patterning, underwater fluorescent devices, sensors, and bioengineering. Inspired by the order of nature, applying soft materials to advance artifcial adaptive coloring will be capable to develop various kinds of practical utility such as sensing, displays, optical flters, anti-counterfeiting, and stealth technologies.

## Experiment

### Materials and measurements

The characterization and experimental materials were treated according to the reported method^17^.

Rhodamine 6G and other main raw chemical were obtained from Aladdin Co. Ltd. All chemical reactants amid experiment reached standard analytical grade or were highly purified without further necessity of purification. The testing species solutions were prepared among distilled water. ^1^H and ^13^C NMR spectra were recorded on a Bruker-AVANCE 400 NMR Spectrometer by ultilizing DMSO-*d*_*6*_ as the solvent and tetramethylsiliane (TMS) as the internal reference. The Fourier transform infrared radiation (FT-IR) spectra of the monomer and hydrogels were recorded with a FT-IR (Thermo fisher Nicolet 6700) instrument by ultilizing an ATR apparatus with 4 cm^−1^ resolution between 4000 and 650 cm^−1^. Element analysis was finished on an Elementar UNICUBE spectrometer. High resolution mass spectra (HRMS) were measured on an Accurate-Mass Q-TOF LC/MS system. UV–Vis absorption spectra were recorded with a Shimadzu UV-2600 spectrophotometer at room temperature. Fluorescence spectra were performed on Hitachi F-4600 fluorescence spectrophotometer using 1 cm path length cuvettes at room temperature.

### Synthesis of R6GLA

**Scheme 1:**
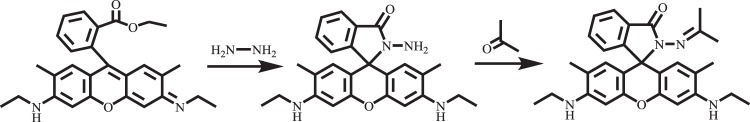
Synthesis of **R6GLA**.

Rhodamine 6G 0.4790 g (1.00 mmol) and 2 mL 80% hydrazine monohydrate were completely dissolved in alcohol(100 mL), among which the mixture was heated until the appearance of reflux for 4.5 hours. Then, the solvent was evaporated, and column chromatography was applied to the resultant residue (dichloromethane/ethanol 40/1, v/v) to generate the intermediate N-(Rhodamine 6 G)lactam (0.3885 g, 0.86 mmol). N-(Rhodamine 6 G)lactam (0.4285 g, 1.00 mmol) and the acetic acid (0.5 mL) were dissolved in 70 mL of acetone and heated to 40 °C, which shall be stirred for 3 hours. When finishing filtering the mixture, the remaining filter cake was rinsed by acetone and purified by column chromatography at the following step(dichloromethane: ethanol = 30/1, v/v) to generate the requisite white solid **R6GLA** (0.3608 g, yield = 77%). The Synthesis of **R6GLA** was recommended in Scheme 1. ^1^H NMR (400 MHz, DMSO-*d*_*6*_, ppm): δ 7.82 – 7.75 (m, 1 H), 7.54 – 7.46 (m, 2 H), 7.00 – 6.93 (m, 1 H), 6.21 (s, 2 H), 6.17 (s, 2 H), 5.01 (t, J = 5.2 Hz, 2 H), 3.18 – 3.02 (m, 4 H), 1.85 (s, 9 H), 1.73 (d, J = 29.0 Hz, 3 H), 1.20 (t, J = 7.1 Hz, 6 H). ^13^C NMR (101 MHz, DMSO-*d*_*6*_, ppm): δ 166.96 (s), 153.48 (s), 151.68 (s), 148.21 (s), 141.34 (s), 131.24 (s), 128.07 (s), 127.32 (s), 125.37 (s), 124.49 (s), 124.13 (s), 122.79 (s), 118.82 (s), 105.13 (s), 96.06 (s), 64.83 (s), 41.88 (s), 37.93 (s), 28.46 (s), 17.46 (s), 14.59 (s). FT-IR: (KBr, cm^–1^) ν 3461 and 2938 (N–H), 1504 (C=N), 1702 (C=O), 1472 (C=C), HRMS (ESI): calculated for: 468.25; Found 469.64 for [M + H]^+^.

### General procedures for spectroscopic analysis of R6GLA

The testing species of metal ions (Na^+^, K^+^, Ca^2+^, Fe^2+^, Fe^3+^, Co^2+^, Ni^2+^, Cu^2+^, Zn^2+^, Pb^2+^, Cd^2+^, Mn^2+^, Ba^2+^, Mg^2+^ and Hg^2+^) were prepared with the density of 1.0 × 10^−3^ mol·L^−1^ in deionized water, and ethylenediamine (EDA) solution was prepared with 1.0 mol·L^−1^ in deionised water. The stock solution (1.0 × 10^−3^ mol·L^−1^) was obtained through dissolving **R6GLA** in DMSO and then was diluted with a mixed solution of DMSO/H_2_O to prepare the analytical solution (DMSO/H_2_O = 7/3, v/v). The pH range adjustment solutions were prepared by 15% HCl solution or 15% NaOH solution.

### Synthesis of hydrogel sensors and bilayer hydrogel sensors

The hydrogel sensor was synthesized according to the reported method^17^.

**Figure 1:**
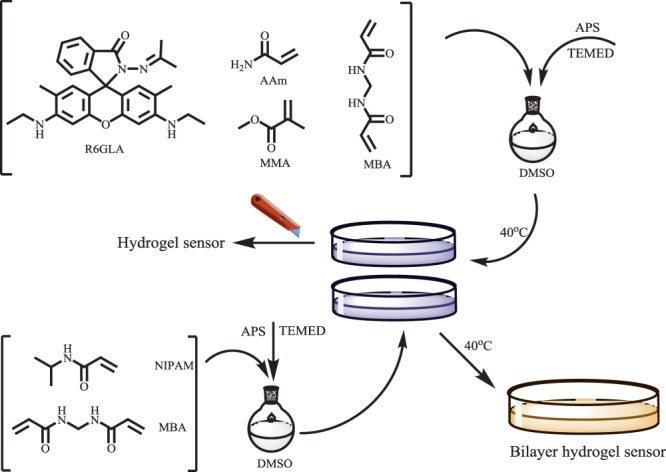
Synthesis of hydrogel sensor and bilayer hydrogel.

The hydrogel sensors were synthesized by free radical polymerization (Fig. 1). AAm, MMA, **R6GLA** and MBA (0.3% of total monomer) were mixed together and dissolved in DMSO solution. This mixture was stirred continuously under high vacuum environment at 15 °C until a homogeneous solution was generated. Then, 100 μL of TEMED and initiator (APS, 2% of total monomer), which were dissolved in 2.0 mL of DMSO were added in the mixture. During the reaction, the mixture was stirred until a homogeneous solution was formed, which was filled into a cylindrical mould in the next step and maintained at 50 °C for 12 hours. Thereafter, the hydrogel sensors were extracted from the mould and washed by DMSO for 36 hours and later by distilled water for 36 hours. One piece of the hydrogels was dried in an oven at 50 °C to study swelling kinetics of hydrogel sensors^17^.

**Figure 2:**
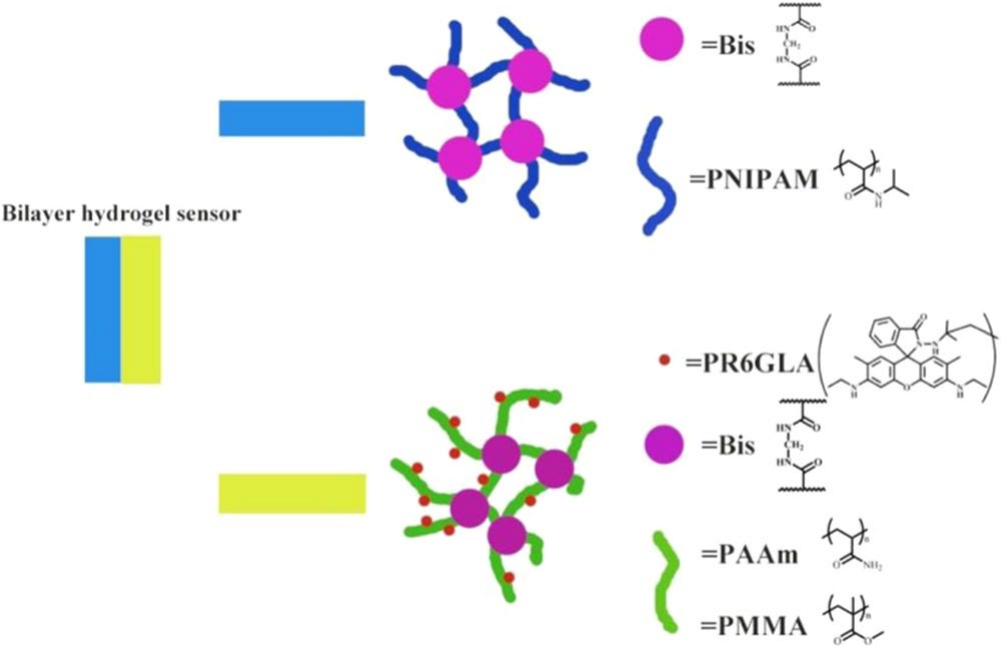
The adhesion of the bilayer hydrogel (PNIPAM hydrogel and hydrogel sensor).

In addition, to synthesize a bilayer hydrogel sensor with shape deformation and fluorescence sensing performance, NIPAM and MBA (0.3% with respect to monomer) were blended reciprocally and dissolved in DMSO solution. Under high vacuum environment, this mixture was stirred at 15 °C until witness the generation of homogeneous solution. Then, 100 μL TEMED and initiator (APS, 2% of total monomer) which were dissolved in 2.0 mL of DMSO, were added in the mixture. During the reaction, the mixture was stirred until the homogeneous solution was generated, which was then poured into a cylindrical mould and maintained at 50 °C for 5 hours. When the mixture solution was almost illiquidity, the DMSO solution, that was dissolved in AAm, MMA, **R6GLA**, MBA (0.3% regarding to monomer) TEMED and initiator (APS, 2% regarding to monomer), was added to the cylindrical mould. The PNIPAM hydrogel was intermingled with the rhodamine-immobilized hydrogel via applying with hydrogel networks-based supramolecular glue to obtain a bilayer hydrogel by interpenetration, as shown in Fig. 2. Thereafter, the bilayer hydrogels were extracted from the mould and rinsed by DMSO for 36 hours and then by distilled water (≤30 °C) for 36 hours.

## Results and discussion

### UV-Vis and fluorescence spectra studies of R6GLA

**Figure 3:**
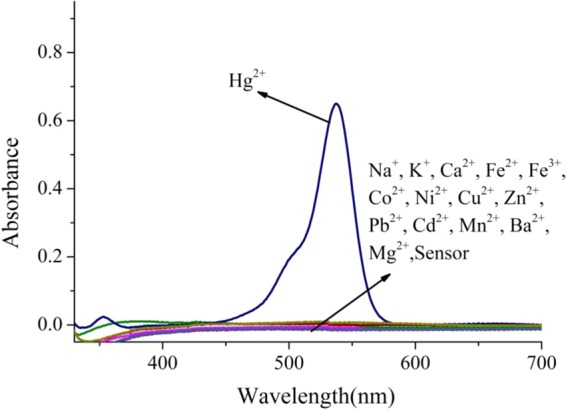
UV–Vis absorption spectral of **R6GLA** in DMSO/H_2_O (7/3, v/v).

The UV–Vis spectras of **R6GLA** toward various metal ions (Na^+^, K^+^, Ca^2+^, Fe^2+^, Fe^3+^, Co^2+^, Ni^2+^, Cu^2+^, Zn^2+^, Pb^2+^, Cd^2+^, Mn^2+^, Ba^2+^, Mg^2+^ and Hg^2+^) were investigated on a UV-Vis spectroscopy, as shown in Fig. 3. Clearly, the solution of **R6GLA** (10^−4^ mol/L^−1^, DMSO/H_2_O, 7/3, v/v) exhibited absorption band at 355‒700 nm through investigation, and adding Hg^2+^ triggered the appearance of an obvious absorption band at 538 nm, which indicated that the **R6GLA** respond selectivly to Hg^2+^. However, absorption band rarely appeared or completely did not appear by adding other metal ions, which proved the prominent selectivity of **R6GLA** toward Hg^2+^ with the possible reason of the complexed property of **R6GLA** toward Hg^2+^ and the photoinduced electron transfer (PET).

**Figure 4:**
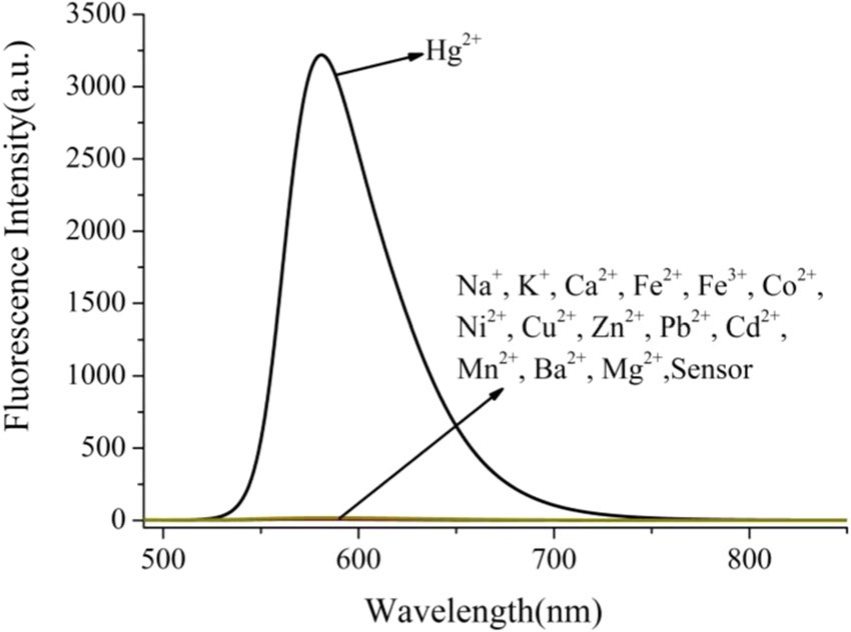
The fluorescence spectra of **R6GLA** in DMSO/H_2_O (7/3, v/v) (λ_ex_ = 490 nm).

**Figure 5:**
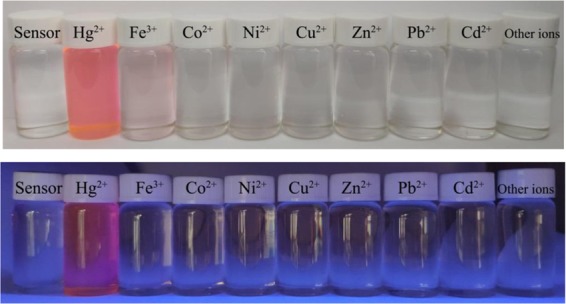
The color changes of **R6GLA** in the presence of different metal ions in DMSO/H_2_O (7/3, v/v) under visible light and UV light at 365 nm.

Fluorescence emission behavior of **R6GLA** toward different metal ions (Na^+^, K^+^, Ca^2+^, Fe^2+^, Fe^3+^, Co^2+^, Ni^2+^, Cu^2+^, Zn^2+^, Pb^2+^, Cd^2+^, Mn^2+^, Ba^2+^, Mg^2+^ and Hg^2+^) was investigated in a solution of DMSO and H_2_O (7/3, v/v) as shown in Fig. 4. The free solution of **R6GLA** in DMSO and H_2_O (7/3, v/v) showed no obvious fluorescence emission, while the addition of Hg^2+^ induced a significant fluorescence enhancement with the significant fluorescence appearance at 581 nm. These results should be ascribed to the blocked photoinduced electron transfer (PET) process and the chelation-enhanced fluorescence (CHEF). In comparison, fluorescence spectra showed no distinct variation by adding other metal ions, which proved the prominent fluorescent selectivity of **R6GLA** toward Hg^2+^ (Fig. 5).

### pH stability studies of R6GLA

**Figure 6:**
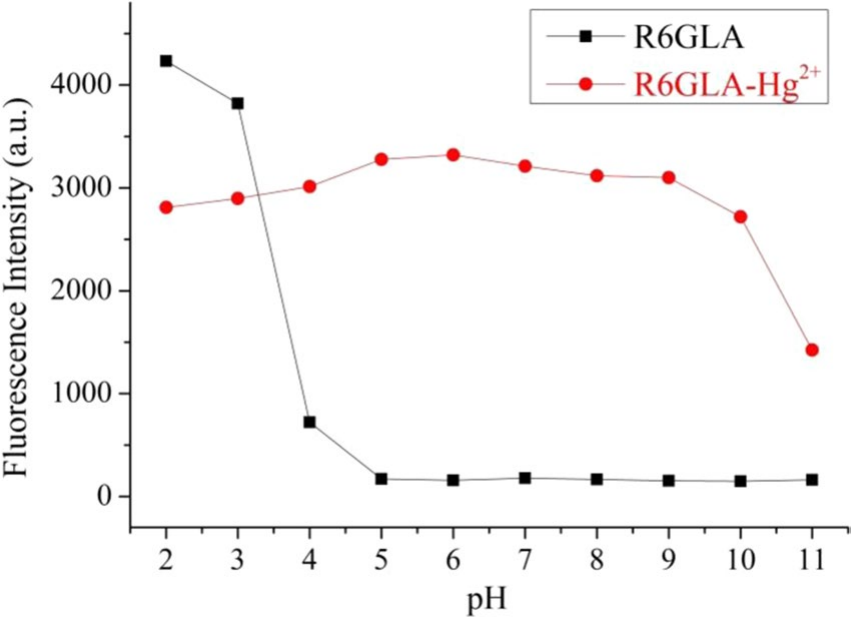
Fluorescence response of **R6GLA** as a function of pH (2.0–11.0) in the absence and presence of Hg^2+^ (λ_ex_ = 490 nm, DMSO/H_2_O = 7/3, v/v).

Aiming at the investigation of the potential future application of the sensor **R6GLA** among diverse environments, it was essential to investigate the pH (in the range of 2.0-11.0) effect on the fluorescence intensity of **R6GLA** and **R6GLA**-Hg^2+^. As shown in Fig. 6, the **R6GLA** remains turning on in a pH range (2.0-4.0), which can be concluded that the intense acidic medium resulted in an significant enhancement in fluorescence appearance. However, The wide range of pH (5.0–10.0) impose no influence on the sensing effectiveness of **R6GLA** towards selective inspection of Hg^2+^. Thus, **R6GLA** is quite convenient in the identification of Hg^2+^ as a fluorescent monomer.

### Reversibility studies of R6GLA

**Figure 7:**
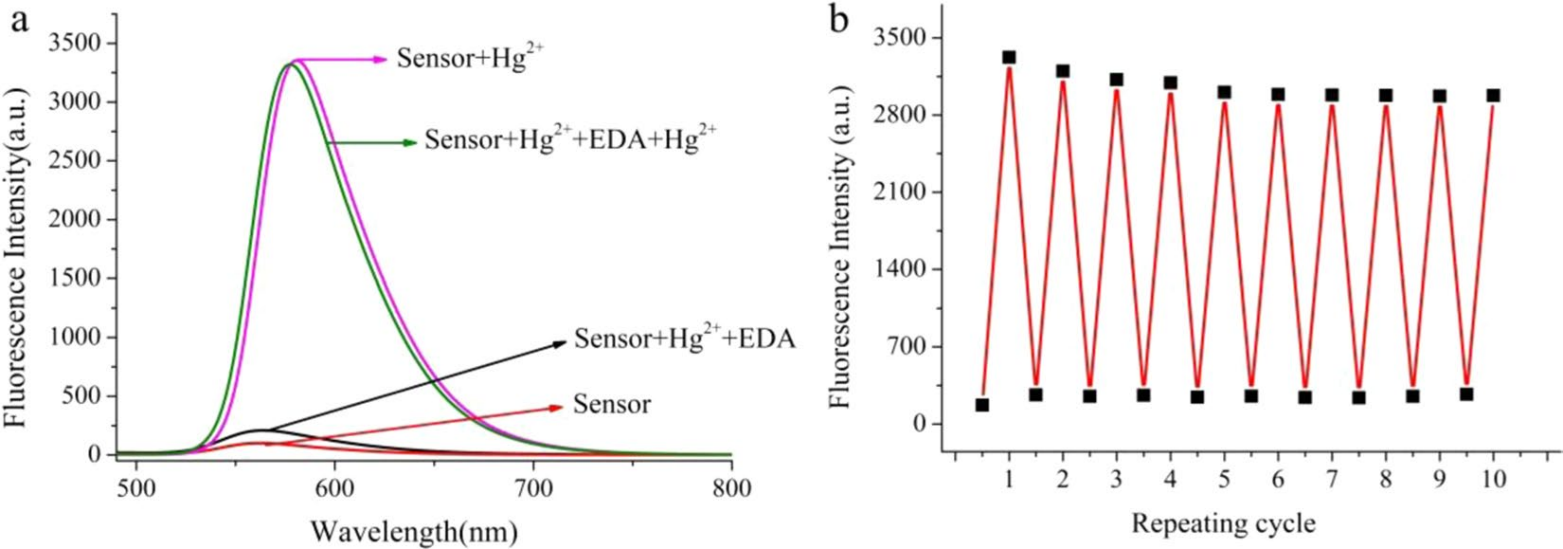
(**a**) Fluorescence reversibility of **R6GLA** (λ_ex_ = 490 nm, DMSO/H_2_O = 7/3, v/v). (**b**) Repeatability of Hg^2+^ sensing behavior of **R6GLA**.

The reversibility of the recognition process of **R6GLA** was performed by adding EDA solution (Fig. 7a). The addition of EDA solution to a mixture of **R6GLA** and Hg^2+^ resulted in the diminution of the fluorescence intensity and led to the recovery of the fluorescence indication of **R6GLA**. Meanwhile, the solution color changed from fluorescent red to transparent, which indicated the regeneration of the free chemosensor **R6GLA**, and the regenerate sensor is still able to respond to Hg^2+^. As shown in Fig. 7b, these results indicated that **R6GLA** could be used as a reversible colorimetric chemosensor for Hg^2+^ in aqueous solution. Such reversibility and regeneration play a vital role for the development of device to effectively sense the Hg^2+^ in aqueous environment.

### Binding stoichiometry and binding sites

**Figure 8:**
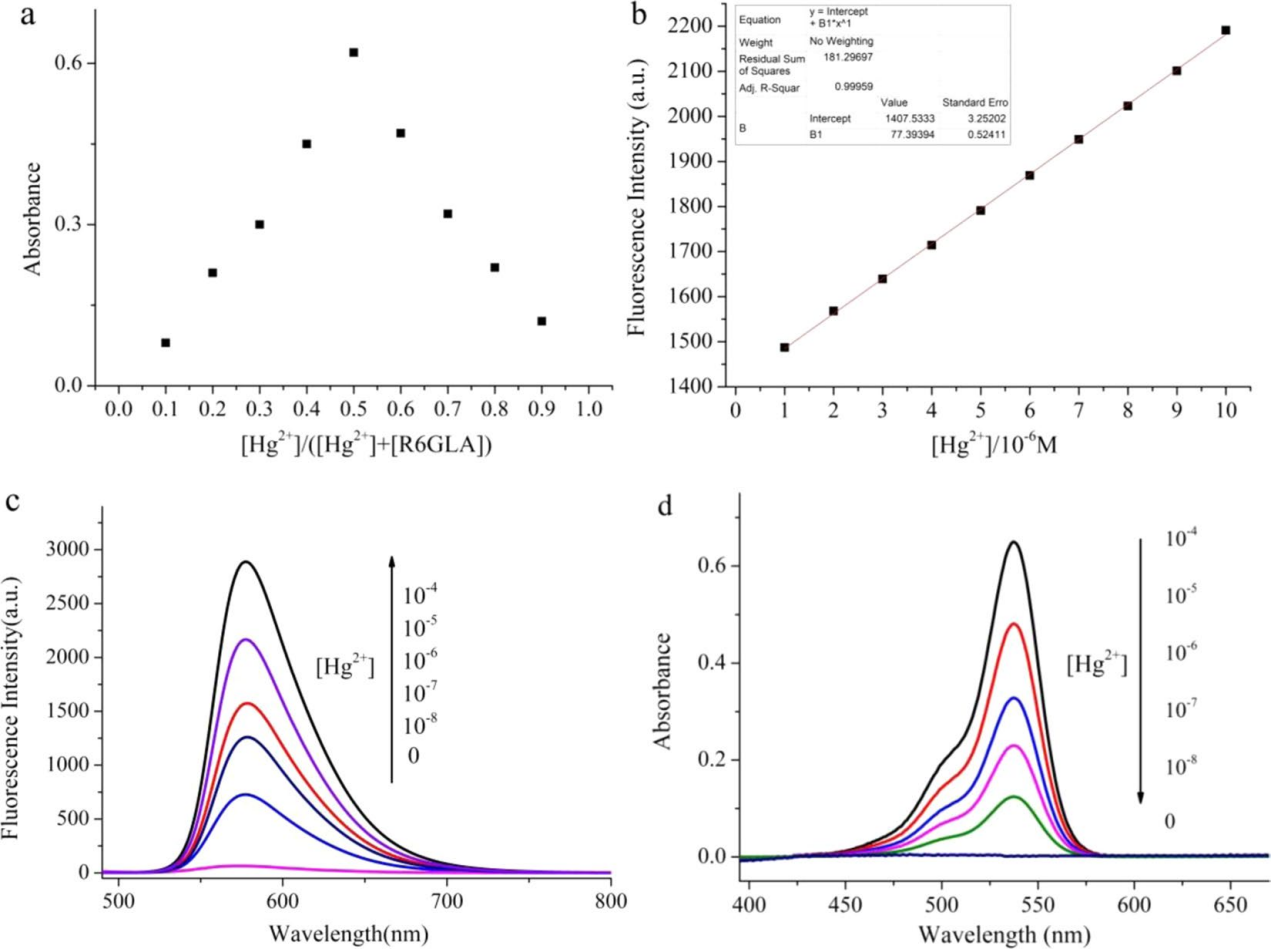
(**a**) Job’s plot for **R6GLA** and Hg^2+^ in DMSO/H_2_O (7/3, v/v) solution, (**b**) calculation of detection limits of **R6GLA** for Hg^2+^ in DMSO/H_2_O (7/3, v/v), (**c**) the fluorescence spectra of **R6GLA** with different concentration of Hg^2+^ in DMSO/H_2_O (7/3, v/v), (**d**) the UV–Vis absorption spectral of **R6GLA** with different concentration of Hg^2+^ in DMSO/H_2_O.

The response of the **R6GLA** to Hg^2+^ was rapid and the fluorescence intensity has been obviously enhanced, which can be attributed to the PET process blocked and CHEF. **R6GLA** displayed very weak fluorescence band at 581 nm, which was due to the quenching by Rhodamine 6 G hydrazide derivatives through a PET. The d orbital of Hg^2+^ was available for forming π bonding and the complex spirolactam ring opening procedure, accompanying with the blocking of PET process. Meanwhile, rigidity of the sensor molecule to produce a CHEF effect by the stable complexation with suppressing the PET process, that resulted in the increasement of fluorescence intensity. The Job’s plot is shown in Fig. 8a. The results demonstrated that 1:1 complex is conducted between **R6GLA** and Hg^2+^. Based on this data, the detection limit (DL) was therefore determined. As displayed in Fig. 8b, the DL obtained from Hg^2+^ was 14.9 nM. The results above indicated that **R6GLA** was highly sensitive to Hg^2+^ and could be used to synthesis fluorescent sensing materials (Fig. 8c).

**Scheme 2:**
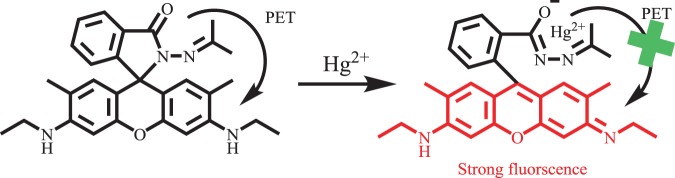
Proposed binding mechanism of **R6GLA** with Hg^2+^.

The proposed binding mechanism was given based upon the Job’s plots and FT-IR spectra investigation as shown in Scheme 2. Free solution of **R6GLA** showed weak fluorescence due to the presence of PET process. However, when Hg^2+^ was added, the PET process was blocked owing to the ring opening of lactam, which resulted in an obvious fluorescence enhancement and visible color variation. The hydrogel sensor proposed binding mechanism was similar to the **R6GLA**.

**Figure 9:**
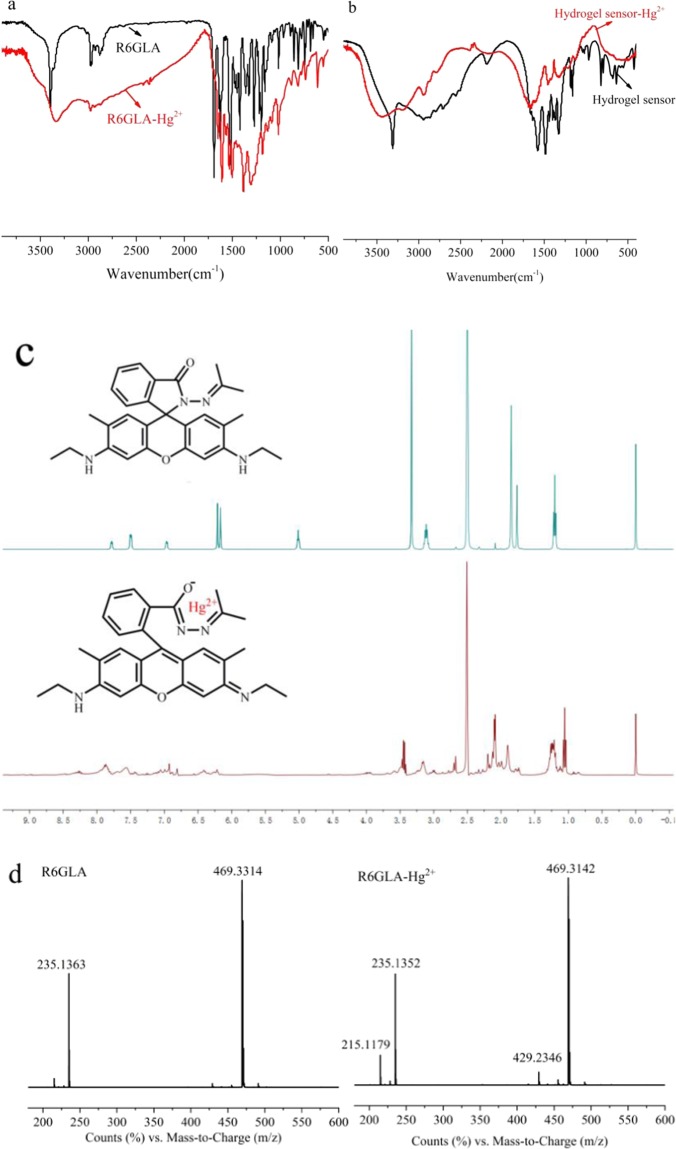
(**a**) FT-IR spectrum of **R6GLA** and **R6GLA**-Hg^2+^ (KBr, cm^−1^) (**b**) FT-IR spectrum of hydrogel sensor and hydrogel sensor-Hg^2+^ (KBr, cm^−1^) (**c**) ^1^H NMR of **R6GLA** and **R6GLA**-Hg^2+^ (400 MHz, DMSO-*d*_*6*_, ppm).

As shown in Fig. 9a, the FT-IR spectra were utilized to study the binding modes of **R6GLA**-Hg^2+^. FT-IR spectrum of **R6GLA** and **R6GLA**-Hg^2+^, among which FT-IR spectrum of hydrogel sensor and hydrogel sensor-Hg^2+^ were shown in and Fig. 9b. The characteristic N-H bands for the compound were observed at 3461 and 2938 cm^−1^. The C=O stretching frequencies of the compound were observed at 1702 cm^−1^ for lactam, simutaneously, the C=N peak was obviously observed at 1504 cm^−1^. The characteristic C=C stretching band for aromatic compounds was observed at 1472 cm^−1^. After chelating with Hg^2+^, the peak of the C=C stretching band of aromatic obviously changed and the C=O peak shifted to 1622 cm^−1^, which supported ring opening of the N-(rhodamine 6G) lactam in **R6GLA**. As shown in Fig. 9c, ^1^H NMR spectra of **R6GLA** and **R6GLA**-Hg^2+^ were investigated (400 MHz, DMSO-*d*_*6*_). Within δ 7.0-8.0 ppm, the **R6GLA** had three kinds of signal. After the addition of Hg^2+^ to **R6GLA**, a new signal peak was observed at 7.79 ppm, which supported that the structure of Rhodamine 6G aromatic ring had changed.

### Colorimetric response behavior of hydrogel sensors

**Figure 10:**
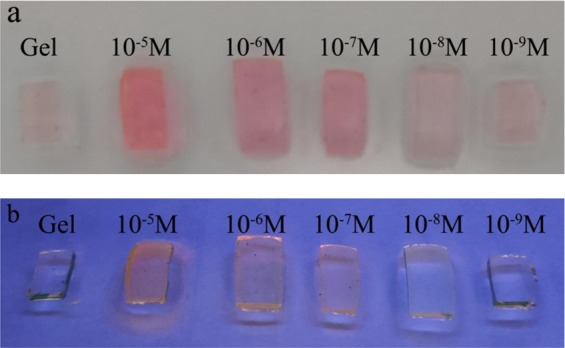
The color of hydrogel sensor in the presence of different concentration of Hg^2+^ under visible light (**a**) and UV light at 365 nm (**b**).

Hydrogel sensor displayed response for Hg^2+^ with high selectivity and sensitivity on the basis of PET and CHEF. To confirm the sensor capability of hydrogel sensor, the detection limits of hydrogels were determined in different concentrations of Hg^2+^ solutions from 10^−3^ mol/L to 10^−8^ mol/L. The color changing of hydrogel sensor still can obviously be observed when the concentration of Hg^2+^ ions was 10^−7^ mol/L. In the measurement of response time, color change began in 20 min for the gel with 3 mm of thickness in 10^−6^ mol/L Hg^2+^ concentration. The color of the gel changed when the concentration of Hg^2+^ was 10^−3^ mol/L, 10^−4^ mol/L and 10^−5^ mol/L in 40 s, 2 min and 10 min respectively. As shown in Fig. 10, in aqueous solution, the hydrogel sensor displayed visible red light, as same as **R6GLA**, and under 365 nm UV light, the color change of hydrogel sensor in aqueous solution was more obvious.

### Reversibility of hydrogel sensors

**Figure 11:**
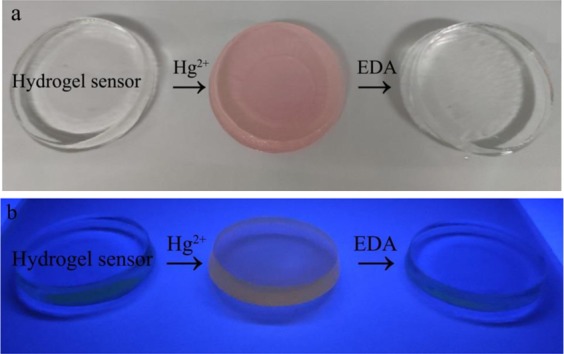
The reuse process with color changes of hydrogel sensors under visible light (**a**) and UV light at 365 nm (**b**).

As shown in Fig. 11, the reusable performance was investigated by putting hydrogel sensor into 1.0 mol·L^−1^ ethylenediamine (EDA) solution. After decolorization and neutralization, hydrogel sensor showed the original color, which was not only regenerated but also still remained the color response performance to Hg^2+^.

### Macroscopic deformation of bilayer hydrogels

**Figure 12:**
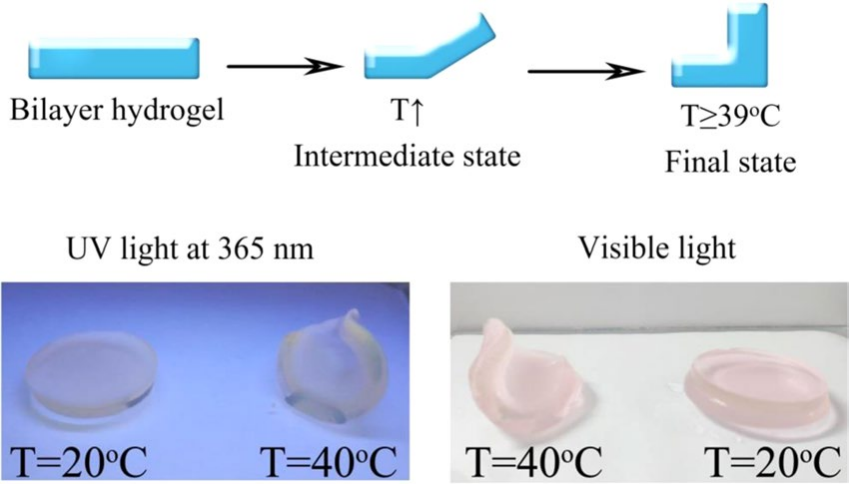
Thermoresponsive bending upon the temperature changing from 20 to 40 °C.

The synthesized bilayer hydrogels showed synergetic shape deformation and Hg^2+^ responsing properties. This bilayer hydrogel can be tailored into various shapes to suit different environment temperature. At low temperature, hydrogel sensor layer and PNIPAM layer were in an unfolding state, among which the PNIPAM sheet showed transparency and has no impact on the excitation light. When the bilayer hydrogel was exposed to warm environment, the PNIPAM layer will shrink and lead to the folding of the hydrogel. The bilayer hydrogel shows original shape below 30 °C. When the environmental temperature increases to 39 °C, it bends toward the PNIPAM side (As shown in Fig. 12). The deformation properties of PNIPAM layer and the responsiveness of Hg^2+^ in the layer of hydrogel sensor are capable to be intergrated without further reciprocal influence, thus the shape deformation and Hg^2+^-sensing characteristic can be integrated into one system.

## Conclusion

In summary, an easy-manipulated and generally applicable strategy to generate an Hg^2+^ responsive hydrogel sensor that is characterized by on-off switchable and color-tunable fluorescence properties has been researched and developed. Deformation and fluorescence color-changing, serves as functions of hydrogel, are integrated and optimized into a same system. The Hg^2+^ responsive hydrogel sensor and the shrunk thermo-responsive deformed PNIPAM hydrogel can be combined to acquire planar sheet that based on the macroscopic supramolecular assembly, then assorted shapes of bilayer hydrogel can be obtained through tailoring. The shape of the bilayer hydrogel could be deformed via thermo-stimulus, and the shape-changing process has no impact on hydrogel sensor sheet fluorescence behavior. Hydrogel sensors have been successfully applied on tracing and amounts identification of Hg^2+^ in aqueous environment, which indicates that hydrogel sensors and bilayer hydrogels satisfies diverse demand from common people to scientists. Additionally, our method has a potential application to fabricate novel intelligent materials and systems that integrates multiple properties, for instance, shape memory, self-adaption, self-healing, and so on. This method provides new insights to profoundly explore smarter biomimetic systems that couples with synergistic visual detection and complex actuating functions.

## Supplementary information

Supplementary information.
