# Supplementary material for: Rhodamine-immobilized optical hydrogels with shape deformation and Hg2+-sensitive fluorescence behaviors
Source: Sci Rep. 2020 May 7;10:7723. doi: 10.1038/s41598-020-64549-5 (PMC7205978; doi:10.1038/s41598-020-64549-5)
Supplement: Supplementary file 1 — Supplementary information. [file 41598_2020_64549_MOESM1_ESM.docx]

**Rhodamine-immobilized optical hydrogels with shape deformation and Hg^2+^-sensitive fluorescence behaviors**

Zixiang Qu^1^, Xia Meng^1^, Hongdong Duan^1, *^, Dawei Qin^1^, Lizhen wang^2^

^1^School of Chemistry and Pharmaceutical Engineering, Qilu University of Technology (Shandong Academy of Sciences), Jinan, Shandong Province, China 250353

^2^Biology Institute, Qilu University of Technology (Shandong Academy of Sciences), Jinan, Shandong Province, China 250014

*Corresponding author.

Tel: +86 13153035598

E-mail address: hdduan67@163.com (H. Duan)

· Image acquisition tools and image processing software packages

· No deliberately obscures manipulations.

The raw images are shown below：

**Fig. 1.** Synthesis of hydrogel sensor and bilayer hydrogel


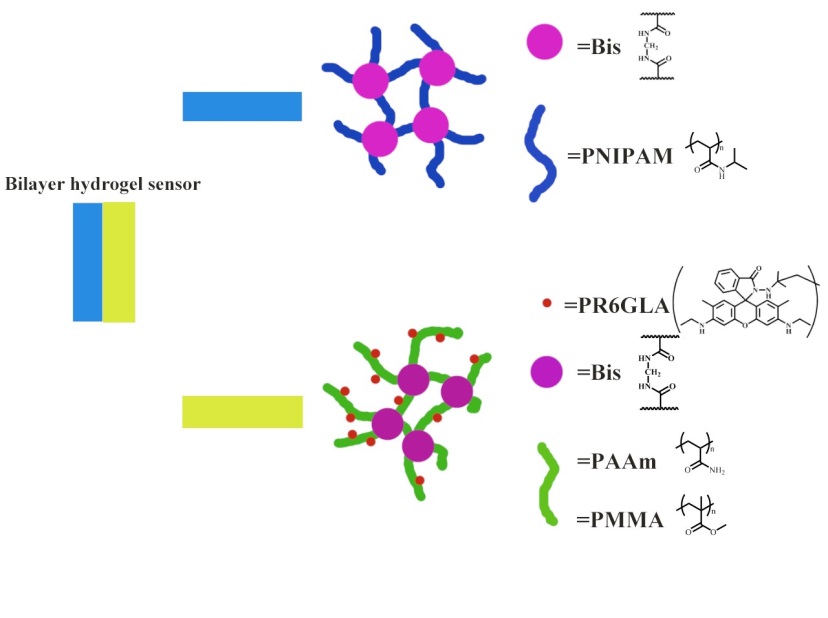


**Fig. 2.** The adhesion of the bilayer hydrogel (PNIPAM hydrogel and hydrogel sensor).


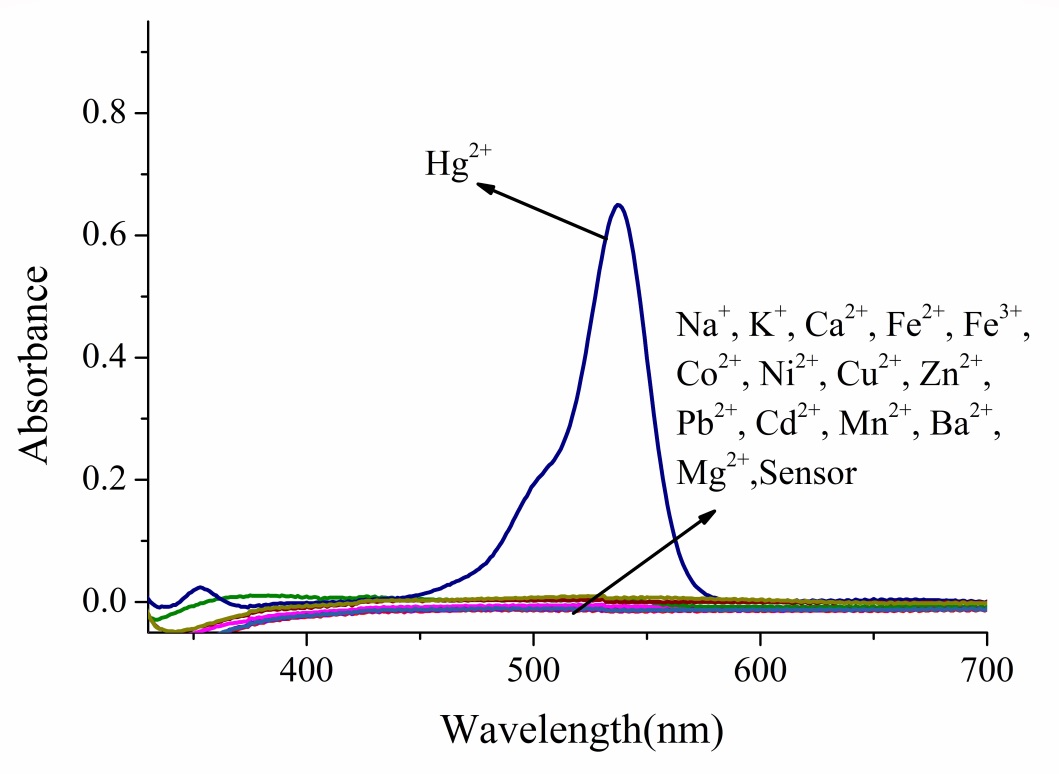


**Fig. 3.** UV–Vis absorption spectral of **R6GLA** in DMSO/H_2_O (7/3, v/v)


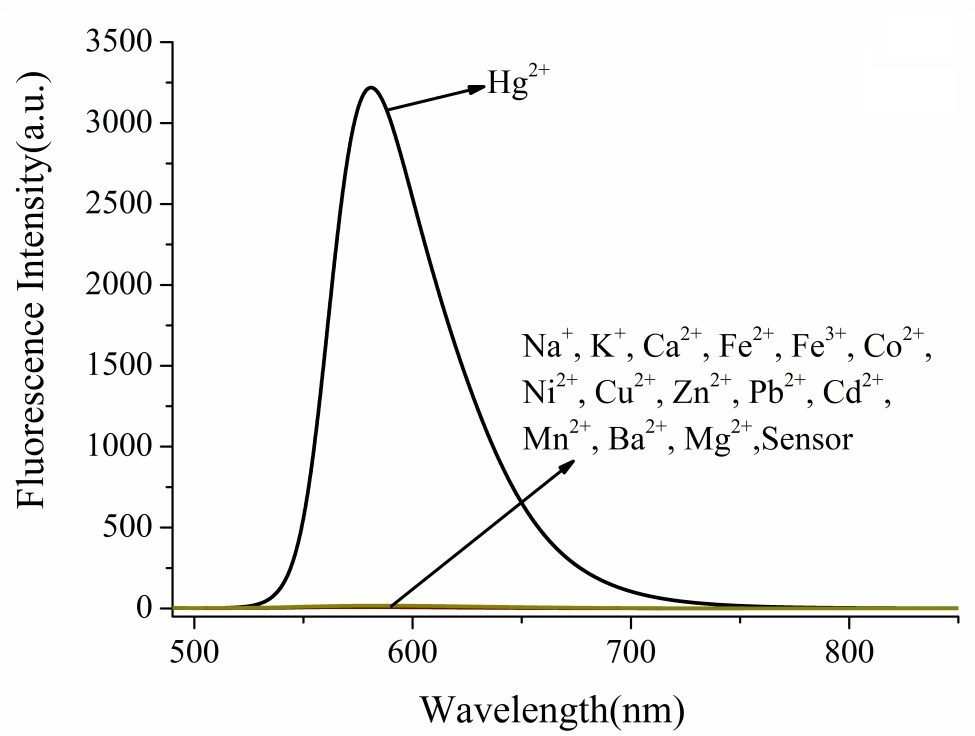


**Fig. 4.** The fluorescence spectra of **R6GLA** in DMSO/H_2_O (7/3, v/v) (λ_ex_=490 nm)


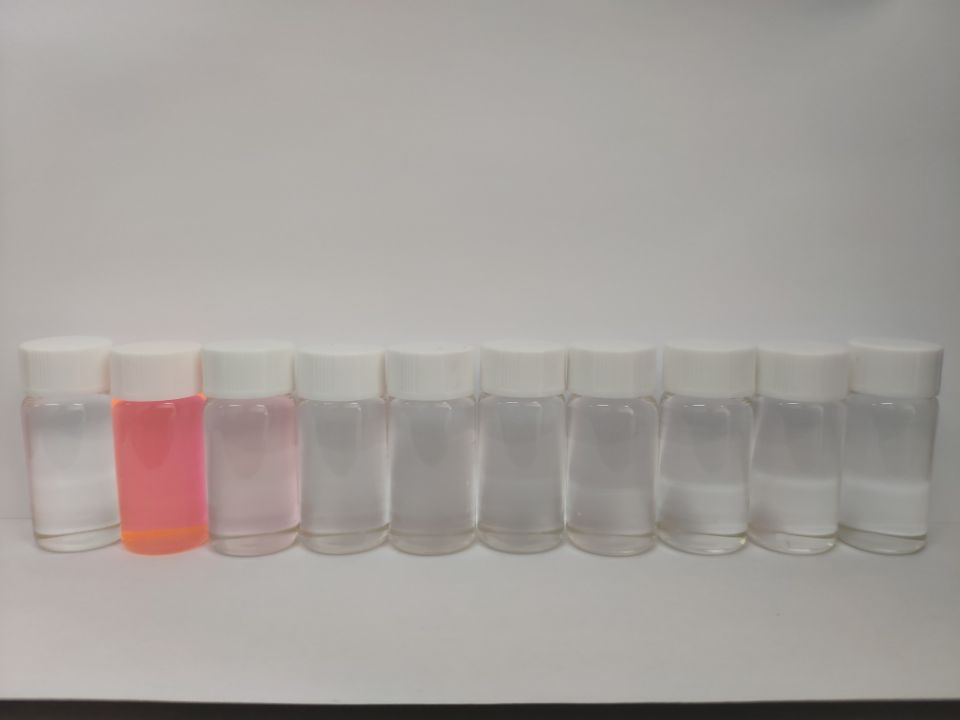


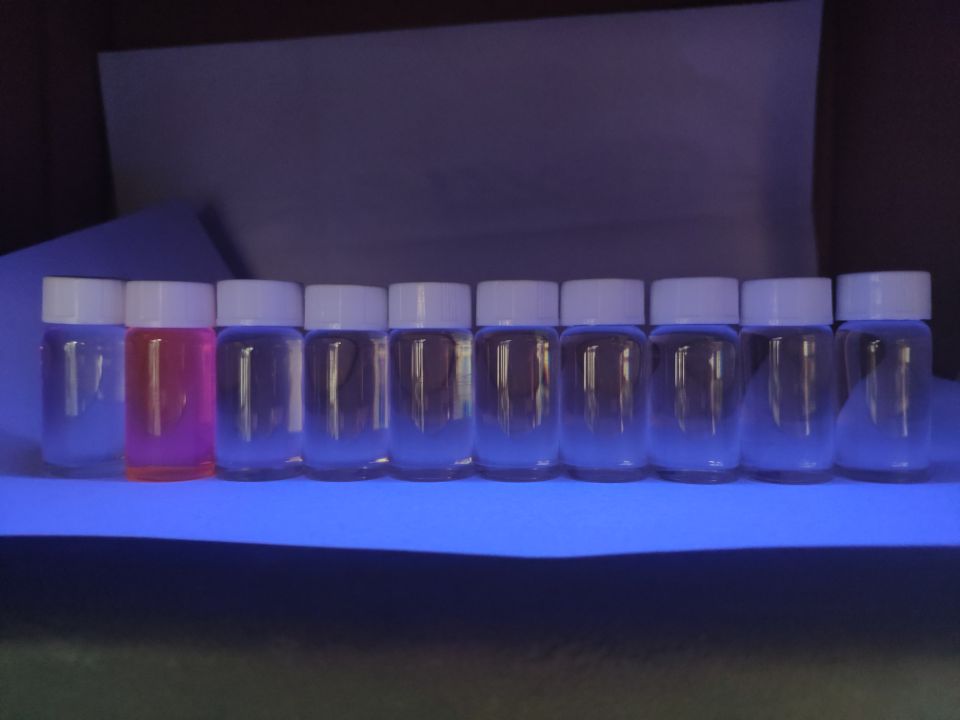


**Fig. 5.** The color changes of **R6GLA** in the presence of different metal ions in DMSO/H_2_O (7/3, v/v) under visible light and UV light at 365 nm.


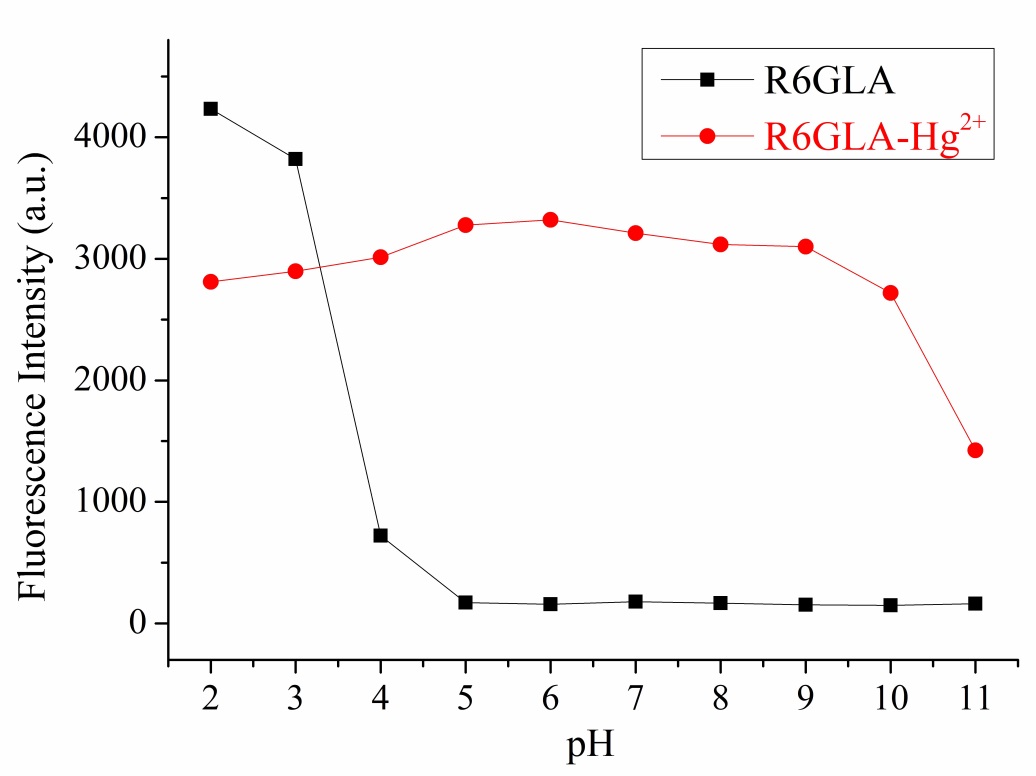


**Fig. 6.** Fluorescence response of **R6GLA** as a function of pH (2.0–11.0) in the absence and presence of Hg^2+^ (λ_ex_ = 490 nm, DMSO/H_2_O=7/3, v/v).


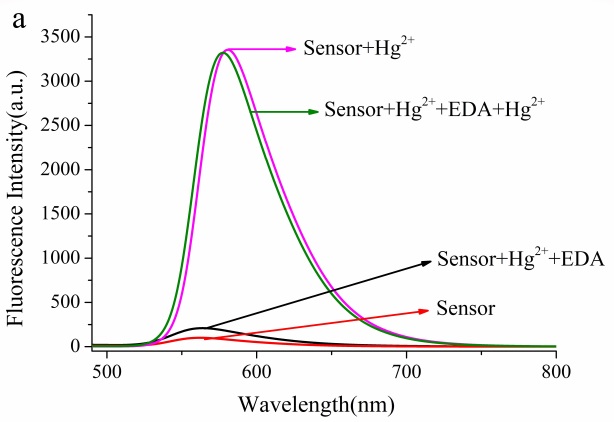

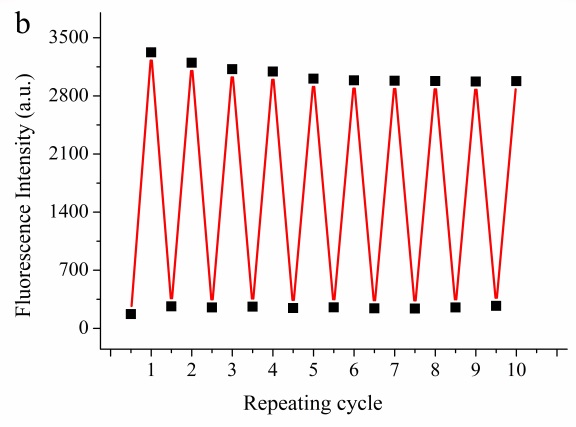


**Fig. 7.** (a) Fluorescence reversibility of **R6GLA** (λ_ex_ = 490 nm, DMSO/H_2_O=7/3, v/v) (b) Repeatability of Hg^2+^ sensing behavior of **R6GLA**


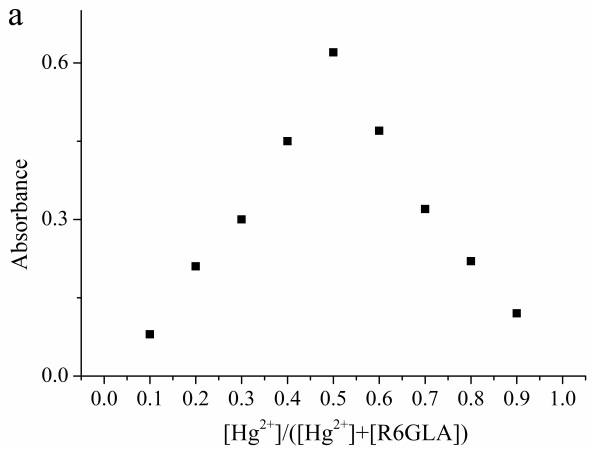

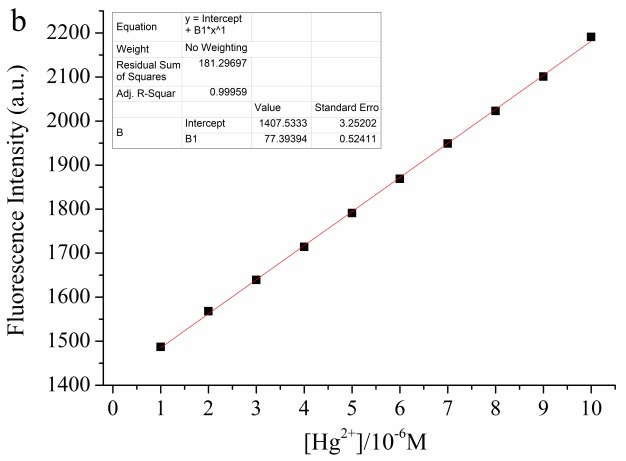

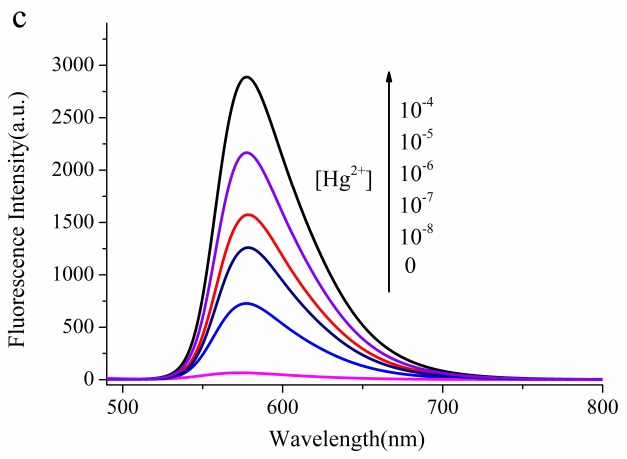

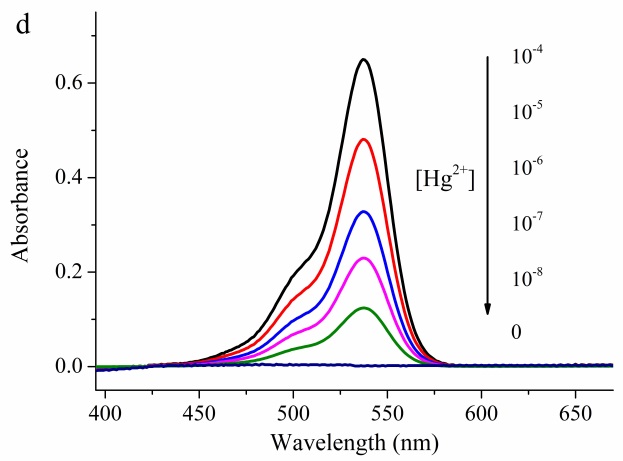


**Fig. 8.** (a) Job’s plot for **R6GLA** and Hg^2+^ in DMSO/H_2_O (7/3, v/v) solution, (b) calculation of detection limits of **R6GLA** for Hg^2+^ in DMSO/H_2_O (7/3, v/v), (c) the fluorescence spectra of **R6GLA** with different concentration of Hg^2+^ in DMSO/H_2_O (7/3, v/v) , (d) the UV–Vis absorption spectral of **R6GLA** with different concentration of Hg^2+^ in DMSO/H_2_O.


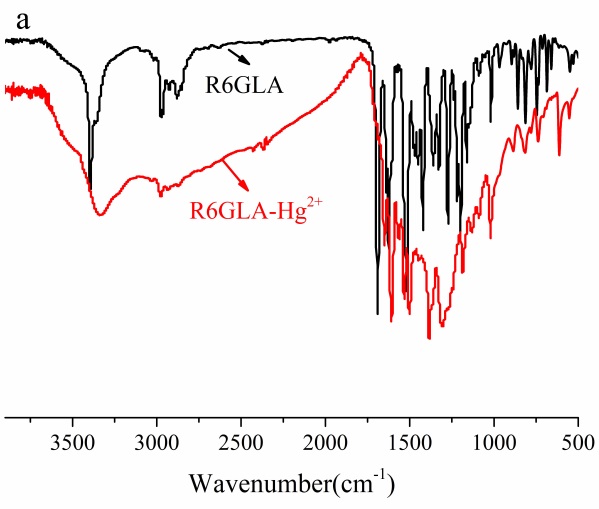

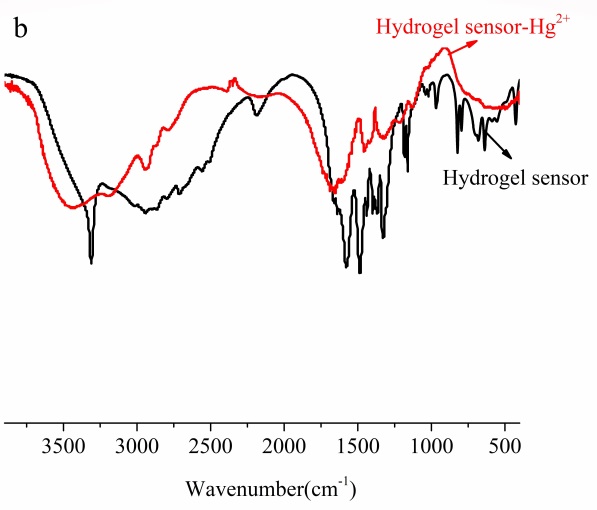


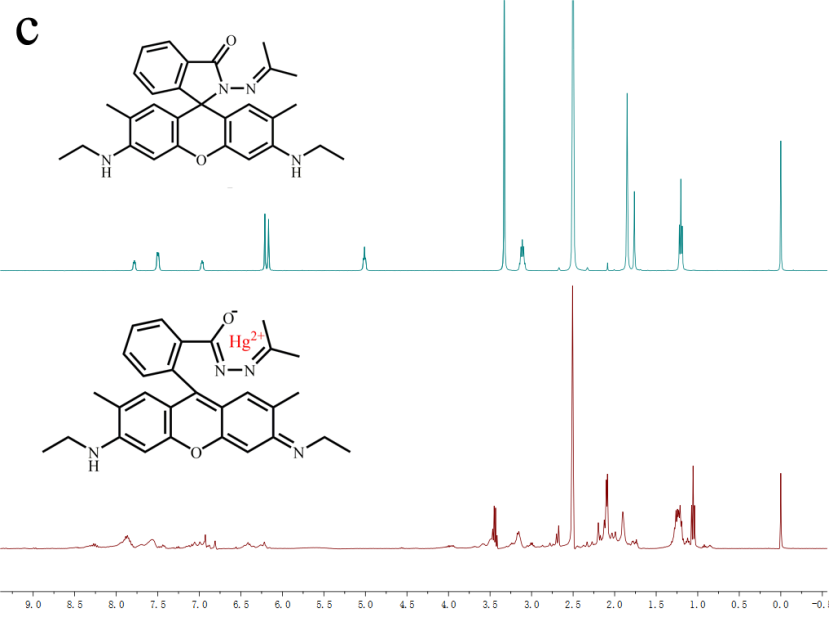


**Fig. 9.** (a) FT-IR spectrum of **R6GLA** and **R6GLA**-Hg^2+^ (KBr, cm^-1^) (b) FT-IR spectrum of hydrogel sensor and hydrogel sensor-Hg^2+^ (KBr, cm^-1^) (c) ^1^H NMR of **R6GLA** and **R6GLA**-Hg^2+^ (400 MHz, DMSO-*d_6_*, ppm)


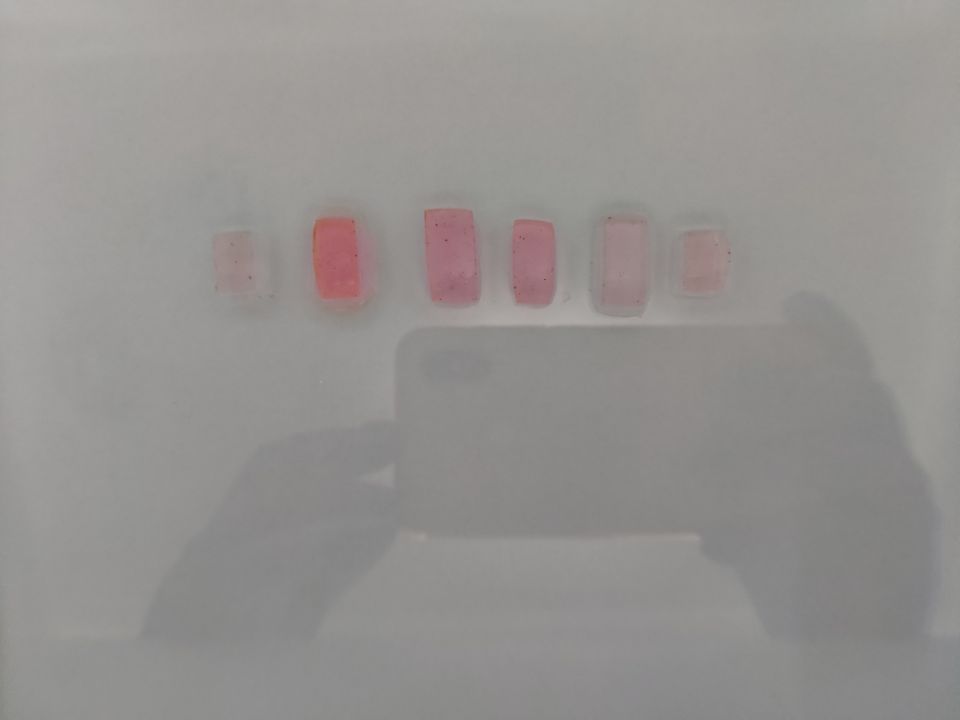


**Fig. 10a.** The color of hydrogel sensor in the presence of different concentration of Hg^2+^ under visible light.


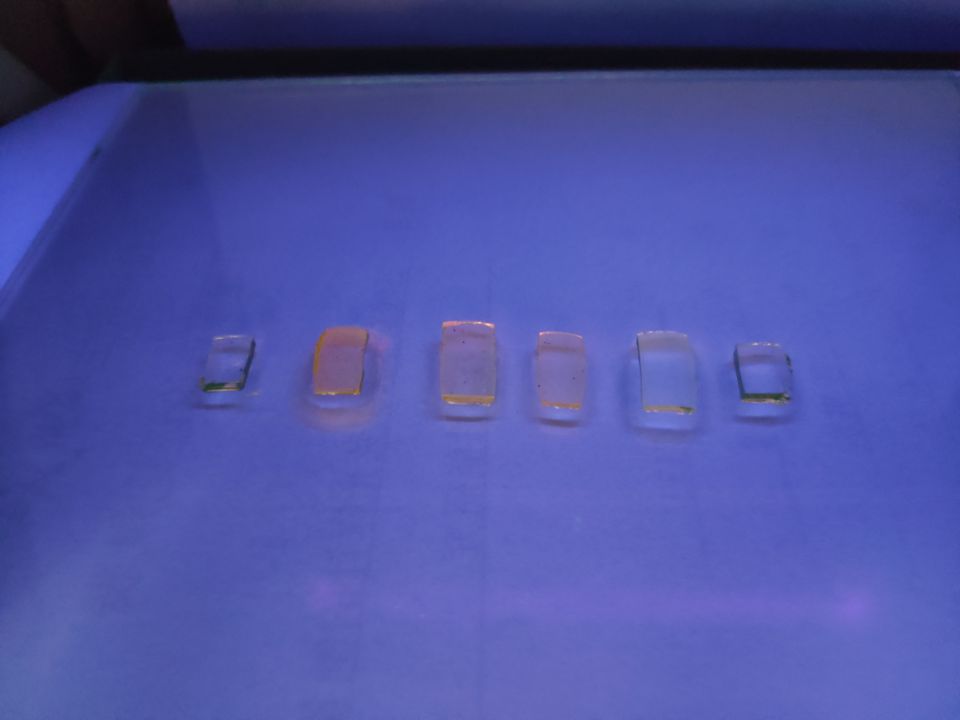


**Fig. 10b.** The color of hydrogel sensor in the presence of different concentration of Hg^2+^ under UV light at 365 nm.


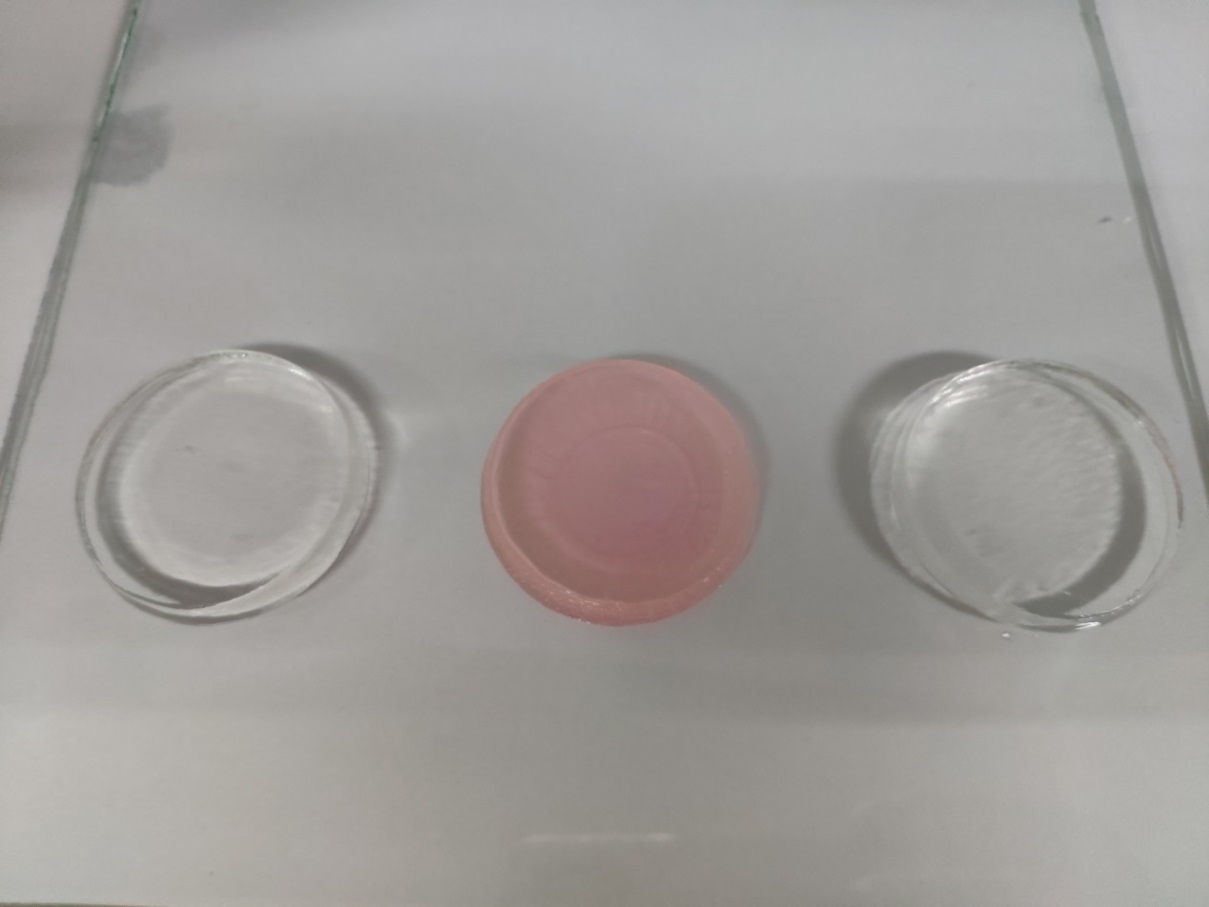


**Fig. 11a.** The reuse process with color changes of hydrogel sensors under visible light.
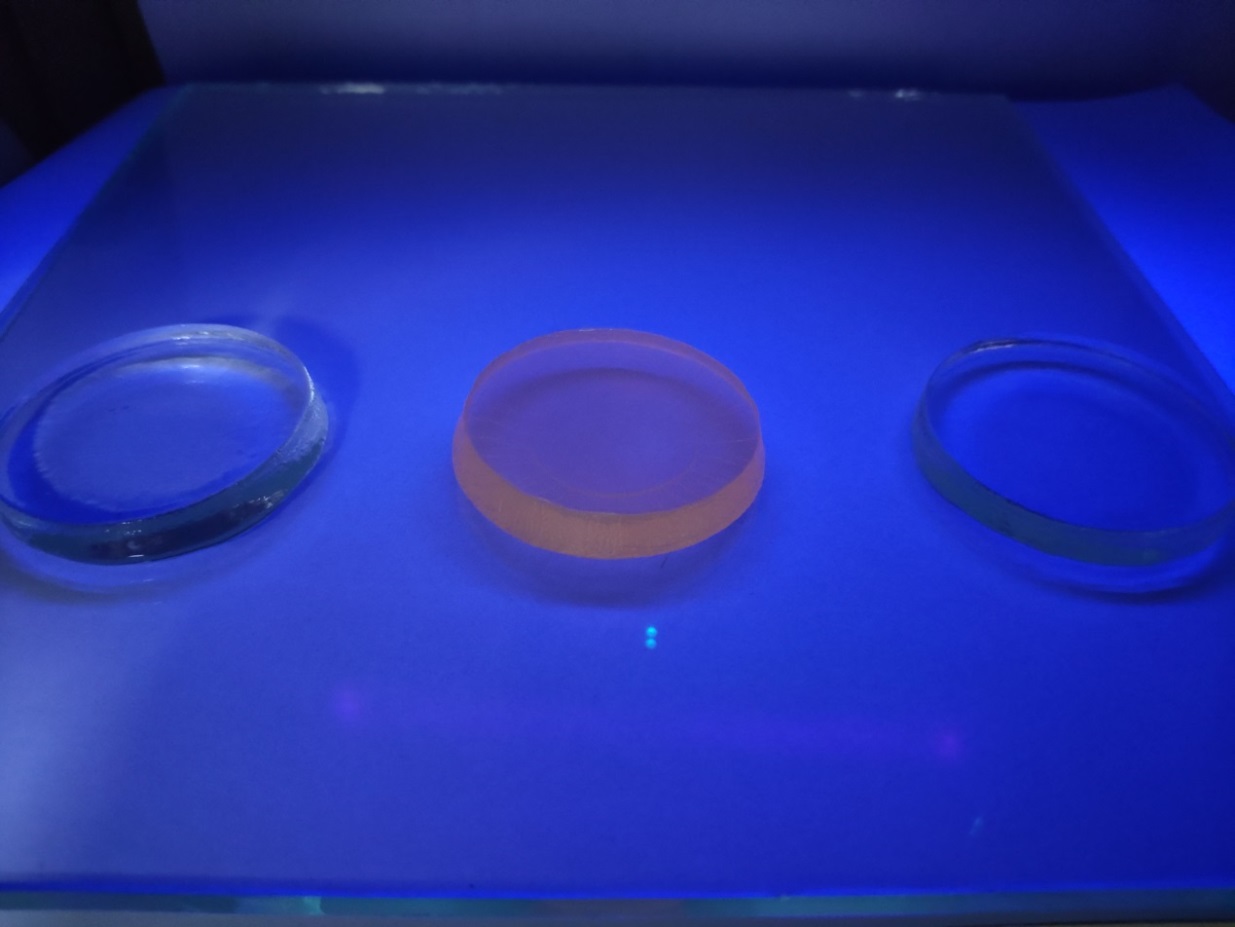


**Fig. 11b.** The reuse process with color changes of hydrogel sensors under UV light at 365 nm.


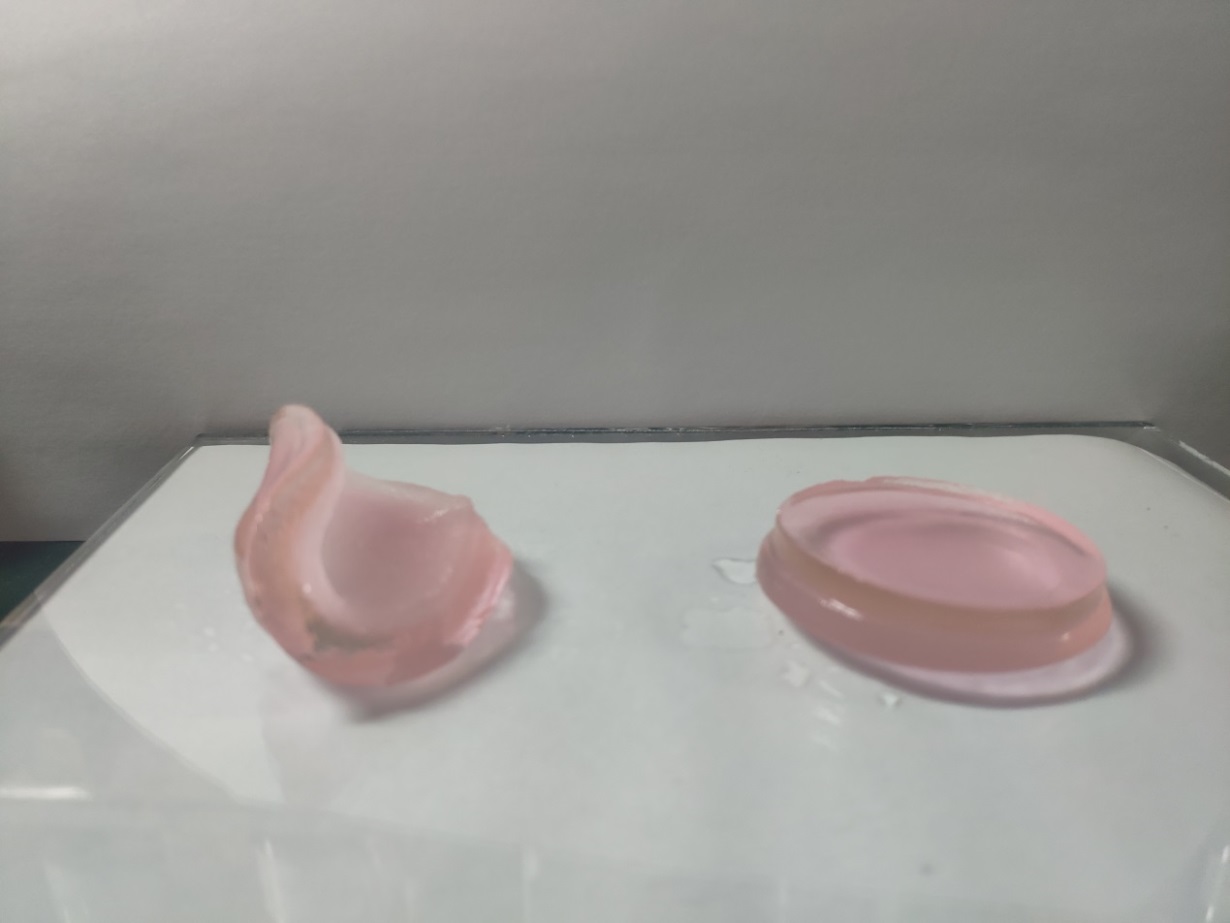


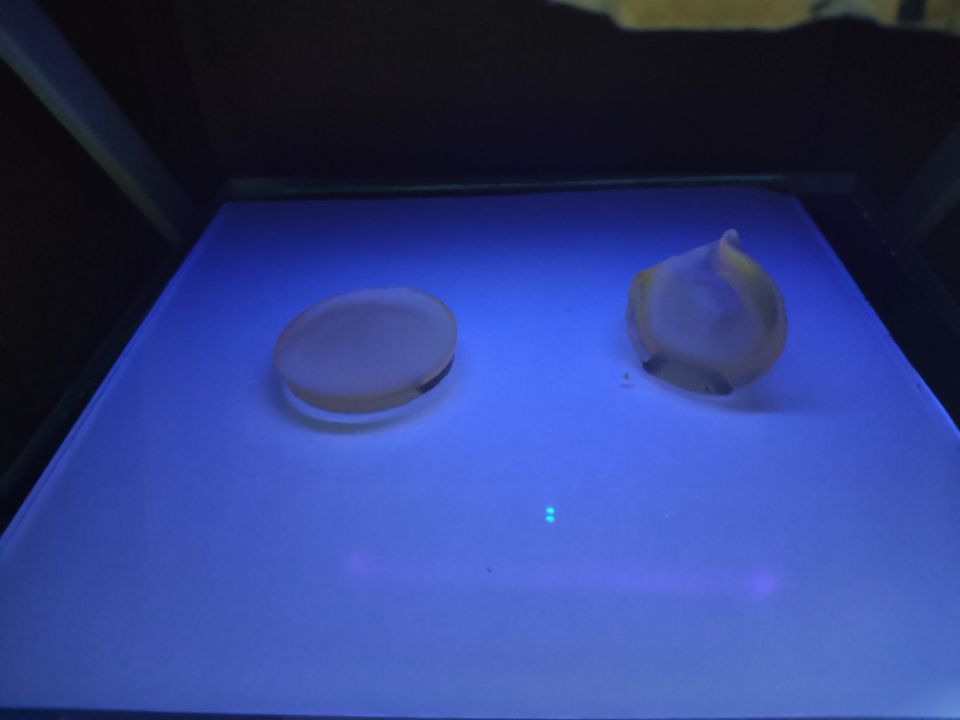


**Fig. 12.** Thermoresponsive bending upon the temperature changing from 20 to 40 °C.
